# Association between serum estradiol levels and abdominal aortic calcification in postmenopausal woman: a cross-sectional study

**DOI:** 10.3389/fendo.2024.1411803

**Published:** 2024-09-20

**Authors:** Lan He, Xu Li, E Shen, Yong-Ming He

**Affiliations:** ^1^ Division of Cardiology, The First Affiliated Hospital of Soochow University, Suzhou, China; ^2^ Department of Ultrasound, Shanghai Chest Hospital, School of Medicine, Shanghai Jiao Tong University, Shanghai, China

**Keywords:** abdominal aortic calcification, estradiol, NHANES, postmenopausal woman, cross-sectional study

## Abstract

**Background:**

The association between Estradiol (E2) levels and abdominal aortic calcification (AAC) in postmenopausal women remains unclear.

**Methods:**

614 postmenopausal women from the 2013-2014 NHANES survey cycle were included in this study. The study population was divided into 3 groups according to E2 tertiles: Tertile1 (2.12-3.57pg/mL), Tertile2 (3.60-7.04pg/mL), and Tertile3 (7.06-38.4pg/mL). Estrogen concentration data were natural logarithmically transformed. A Kauppila score > 5 was regarded as prominent arterial calcification and was used to define (EAAC). Logistic regression models were used to assess the association between E2 levels and EAAC prevalence. Subgroup analyses were performed to test whether the association between E2 levels and EAAC prevalence was consistent in different groups. Sensitivity analyses tested the stability of the model in women older than 45 years.

**Results:**

EAAC prevalence was significantly higher in Tertile1 (16.6%) than in Tertile2 (9.8%) and Tertile3 (8.3%). On a continuous scale, the adjusted model showed a 58% [OR (95%CI), 1.58 (1.02, 2.54)] increase in the risk of EAAC prevalence for per unit decrease in ln(E2). On a categorical scale, the adjusted model showed that Tertile1 and Tertile2 were 2.55 [OR (95%CI), 2.55 (1.10, 5.92)] and 1.31[OR (95%CI), 1.31(1.03, 2.57)] times higher risk of suffering from EAAC than Tertile3, respectively.

**Conclusion:**

This study found that a higher prevalence of AAC in postmenopausal women is closely associated with lower serum E2 levels. Our research further underscores the importance of E2 in maintaining cardiovascular health in postmenopausal women and suggests that monitoring E2 levels may aid in the early prevention and management of AAC and related cardiovascular diseases.

## Introduction

Postmenopausal women often experience a range of menopausal symptoms, primarily including hot flashes, night sweats, sleep disturbances, and genitourinary discomfort (1). Other common symptoms and conditions include bone loss, obesity, low libido, mood swings, and cognitive changes (2). These effects are largely attributed to the decline in estrogen levels (3). Estradiol (E2) is a female hormone and the most biologically active form of estrogen. It is primarily secreted by the ovaries, but also by the adrenal glands, fat tissue, and liver, and it circulates in the bloodstream (4). Numerous studies have highlighted the benefits of E2 for the human body, including the prevention of bone loss, protection against ischemia-reperfusion injury, relief of menopausal symptoms, prevention of neuronal degeneration, maintenance of vaginal health, and assistance in treating ovarian failure or hypogonadism. Over the years, a significant amount of anecdotal evidence has accumulated, supporting the view that postmenopausal E2 may reduce the risk of cardiovascular disease (5).

Abdominal aortic calcification (AAC) is the process in which calcium and phosphate-based minerals deposit in the walls of the abdominal aorta. Driven by various risk factors such as aging, prolonged dialysis, osteoporosis, diabetes, and hypertension, the aortic walls undergo irreversible calcification and become brittle (6). This condition is a significant warning sign for cardiovascular disease. AAC is an important predictor of cardiovascular disease risk, including conditions such as carotid atherosclerosis, myocardial infarction, congestive heart failure, stroke, and intermittent claudication (7–10). It is also a significant indicator of both all-cause mortality and cardiovascular mortality (11). AAC can be easily assessed and semi-quantified using lateral X-rays of the lumbar spine and lateral scans from DXA (Dual-energy X-ray Absorptiometry) (12). Consequently, in recent years, AAC’s role in predicting cardiovascular risk has garnered increasing attention.

The specific mechanisms linking the decline in estradiol levels to increased cardiovascular risk, particularly in relation to AAC, remain unclear. Previous studies have indicated that the incidence and severity of AAC are significantly higher in postmenopausal women (13–16). This study aims to explore the relationship between E2 levels and AAC in postmenopausal women, which could provide valuable insights for the prevention and management of cardiovascular disease in this population.

## Methods

### Study population

The subjects for this study were selected from the National Health and Nutrition Examination Survey (NHANES). NHNES is a major program of the National Center for Health Statistics that uses stratified and probability sampling principles to select a representative portion of the U.S. non-institutionalized population for the purpose of assessing their health and nutritional status. The NHANES survey protocol was approved by the National Institutes of Health Research Ethics Review Board prior to implementation of the survey, and all participants signed and provided informed consent. The absence of regular menstruation for the past 12 months was considered a postmenopausal state. Exclusion criteria for study population: i) Male; ii) Age < 45; iii) Regular menstruation for the past 12 months; iii) Irregular menstruation due to hysterectomy or other reasons; and iii) Missing data on menstruation, AAC and E2. The study population was divided into 3 groups according to E2 tertiles: Tertile1(2.12-3.57pg/mL), Tertile2 (3.60-7.04pg/mL), and Tertile3 (7.06-38.4pg/mL).

### Natural logarithmic conversion of E2

In this study, we explored the relationship between estrogen levels and vascular calcification. The estrogen level data exhibited a significant skewed distribution on the original scale. To meet the normality assumption required for statistical analysis and to mitigate the effects of skewness, we applied a natural logarithmic transformation to the estrogen concentration data.

Specifically, the estrogen concentration (measured in pg/mL) was log-transformed using the following formula:


ln(E2)=ln(E2,pg/mL)

where E2 represents the serum estrogen concentration, and ln denotes the natural logarithm. The log-transformed variable ln(E2) was subsequently used in the statistical analysis.

### Definition and measurement of exposure, study outcome and covariates

The outcome of this study was the prevalence of extensive AAC (EAAC). The degree and extent of AAC was assessed using the AAC-24 semiquantitative technique (Kauppila 1997) (17). A Kauppila score > 5 was regarded as prominent arterial calcification and was used to define EAAC (18–21). Age, Sex, and Ethnicity were obtained from the demographics of the National Center for Health Statistics.

Smoking, Drinking, Hypertension, and Diabetes Mellitus (DM) were obtained through questionnaires. The definitions of smoking and drinking referred to the latest standards on the New Zealand Ministry of Health website (22). Hypertension was defined as self-reported by asking the question, “Have you been told by a doctor that you have hypertension?”. Osteoporosis was defined by self-reported question “Ever told had osteoporosis?”. Osteoporosis prescription drugs were defined by self-reported question “If diagnosed with osteoporosis, whether the participant has been treated by prescription medicine (Fosamax, Boniva, Actonel, Reclast, Miacalcin, Fortical, Evista or Forteo)”. DM diagnosis referred to the most recent ADA criteria (FPG≥7.0 mmol/L or A1C ≥ 6.5% or 2-h OGTT ≥11.1mmol/L or a random plasma glucose≥11.1 mmol/L) (23). Body mass index (BMI) was obtained by physical examination, where BMI was evaluated by body mass (kilograms) and body height (m^2^). Red blood cell (RBC), White blood cell (WBC), Platelets, Albumin, Creatinine (Cr), Triglycerides (TG), Low-density lipoprotein cholesterol (LDL-C), High-density lipoprotein cholesterol (HDL-C) were obtained by laboratory measurements. Blood cell counts and were analyzed with a Beckman Coulter MAXM or DXH 800. Albumin and Cr were measured using the DcX800 method. TG and HDL-C were analyzed by the Roche/Hitachi Modular P Chemistry Analyzer (Mod P) in Mobile Examination Centers (MECs). LDL-C was calculated from measured values of TG, HDL-C, and total cholesterol according to the Friedewald calculation (24). Measurements of E2 in serum are performed using isotope dilution liquid chromatography tandem mass spectrometry (ID-LC-MS/MS) method for routine analysis developed by CDC. Details on the methods are publicly available on the official NHANES website (25).

### Statistical analyses

The Shapiro-Wilk test was used to examine the normality of the data. The Cochran-Armitage test was used to test for between-group trends. Logistic regression model was used to assess the association between E2 levels and EAAC prevalence. The multivariate model was adjusted for Age, Sex, Ethnicity, BMI, Drinking, Smoking, DM, Hypertension, RBC, WBC, Platelets, Albumin, Cr, TG, LDL-C, and HDL-C. Subgroup analyses were performed to test whether the association between E2 levels and EAAC prevalence was consistent in different groups. Continuous variables in subgroups were grouped by median. Sensitivity analyses tested the stability of the model in women older than 45 years. To handle missing data in our analysis, we employed the multiple imputation (MI) method. Specifically, we used the Stata 17 (Stata Corp, TX, US) to perform the imputations. This approach involves creating multiple datasets (25 times in our case) where the missing values are replaced by plausible data points estimated based on observed data patterns. The imputation model included all relevant covariates to account for potential correlations with the missing data. After creating the imputed datasets, we combined the results from each dataset using Rubin’s rules to obtain the final estimates and confidence intervals. All tests were two-sided. Statistical significance was considered when a *P* < 0.05.

## Results

### Baseline characteristics

**Figure 1:**
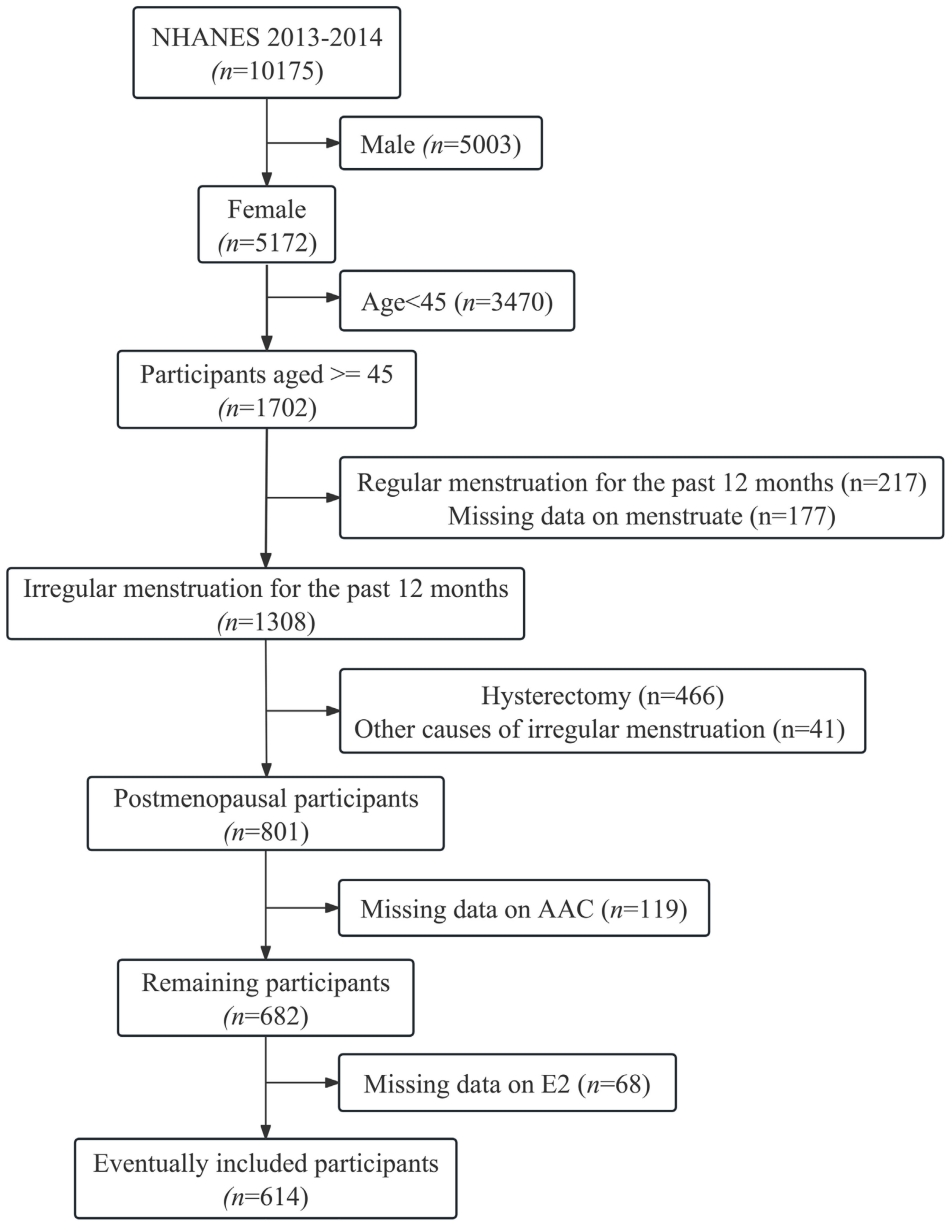
Flowchart for inclusion of participants. E2, Estradiol; AAC, abdominal aortic calcification.

**Figure 2:**
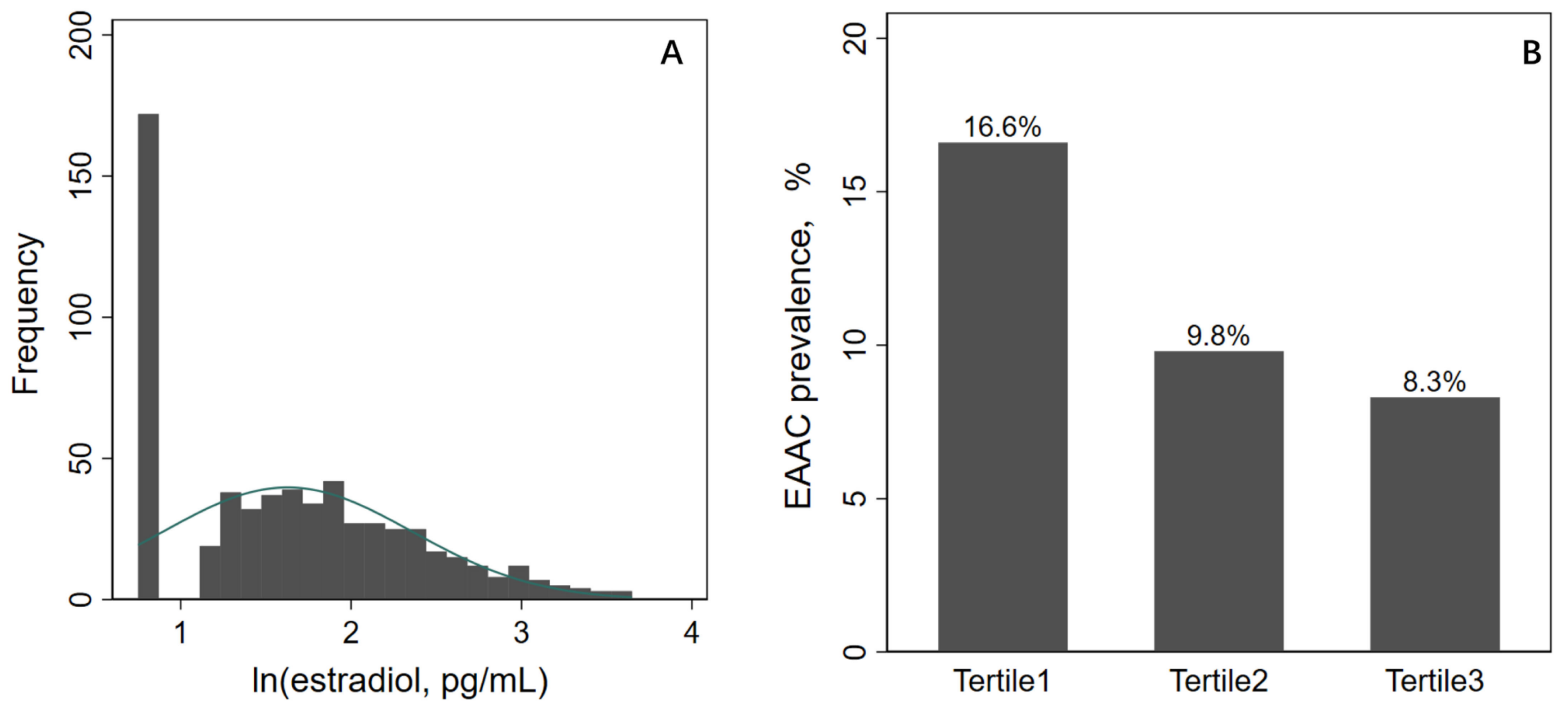
ln(E2) distribution and EAAC prevalence. E2, estradiol; AAC, abdominal aortic calcification.

A total of 10,175 participants were included in the potential analyses during the 2013-2014 NHANES survey cycle, and 614 postmenopausal women were ultimately enrolled in this study after exclusion of participants who were male, older than 45 years, had regular menstruation or irregular menstruation not due to menopause, and had missing AAC and E2 data (**Figure 1**). ln(E2) levels showed a normal distribution, and EAAC prevalence was significantly higher in Tertile1 (16.6%) than in Tertile2 (9.8%) and Tertile3 (8.3%) (**Figure 2**). In addition, participants in the lower tertile were older, more likely to have diabetes mellitus, had higher TG levels and lower HDL-C levels (**Table 1**).

**Table 1:** Baseline characteristics stratified by E2 tertiles.

Factor	Tertile1	Tertile2	Tertile3	*P* for trend
N	205	205	204	
E2, pg/mL	2.31 ± 0.45	5.16 ± 1.03	17.43 ± 21.92	0.000
ln (E2)	0.82 ± 0.17	1.62 ± 0.20	2.59 ± 0.59	0.001
Age, year	65.56 ± 9.84	64.87 ± 8.40	62.43 ± 9.10	0.006
Ethnicity				0.349
Mexican American	39 (19.0%)	22 (10.7%)	19 (9.4%)	
Non-Hispanic White	85 (41.5%)	108 (52.7%)	94 (46.5%)	
Non-Hispanic Black	23 (11.2%)	23 (11.2%)	45 (22.3%)	
Non-Hispanic Asian	29 (14.1%)	26 (12.7%)	16 (7.9%)	
Others	29 (14.1%)	26 (12.7%)	28 (13.9%)	
Drinking				0.929
Never	54 (26.3%)	53 (25.9%)	53 (26.2%)	
Former	45 (22.0%)	37 (18.0%)	41 (20.3%)	
Current	106 (51.7%)	115 (56.1%)	108 (53.5%)	
Smoking				0.859
Never	130 (63.4%)	132 (64.4%)	122 (60.4%)	
Former	43 (21.0%)	46 (22.4%)	50 (24.8%)	
Current	32 (15.6%)	27 (13.2%)	30 (14.9%)	
DM	43 (21.0%)	49 (23.9%)	62 (30.7%)	0.024
Hypertension	54 (26.3%)	51 (24.9%)	61 (30.2%)	0.383
Osteoporosis	48 (23.4%)	40 (19.6%)	28 (13.7%)	0.042
OPD	32 (68%)	27 (69%)	11 (39%)	0.022
BMI, kg/m²	25.208 ± 4.52	28.47 ± 5.49	32.08 ± 6.17	0.000
RBC, 10³/μL	4.33± 0.41	4.43 ± 0.36	4.52 ± 0.40	0.000
WBC, 10³/μL	6.62 ± 1.85	6.83 ± 1.88	7.13 ± 2.00	0.003
Platelets, 10³/μL	238.37 ± 56.33	236.98 ± 56.31	239.35 ± 56.77	0.751
Albumin, g/L	42.12 ± 2.85	42.05± 2.61	41.15 ± 2.94	0.010
Cr, umol/L	71.42 ± 22.75	71.88 ± 20.84	74.59 ± 37.93	0.651
TG, mmol/L	1.26 ± 0.65	1.37 ± 0.78	1.42 ± 0.70	0.010
LDL-C, mmol/L	2.99 ± 0.89	3.05 ± 0.79	3.17 ± 0.98	0.059
HDL-C, mmol/L	1.63 ± 0.47	1.51 ± 0.43	1.48 ± 0.43	0.001
EAAC	34 (16.6%)	20 (9.8%)	17 (8.3%)	0.009

Continuous and categorical variables were presented as mean ± SD or percentages n (%), respectively. E2, estradiol; DM, diabetes mellitus; OPD, osteoporosis prescription drugs; BMI, body mass index; RBC, red blood cell; WBC, white blood cell; Cr, Creatinine; TG, triglycerides; LDL-C, low-density lipoprotein cholesterol; HDL-C, high-density lipoprotein cholesterol; EAAC, extensive abdominal aortic calcification.

### Association between E2 and EAAC

On a continuous scale, the crude model showed a 54% [OR (95%CI), 1.54(1.09, 2.18)] increase in the risk of EAAC prevalence for per unit decrease in ln(E2), which was a 58% [OR (95%CI), 1.58(1.02, 2.54)] increase after adjusting for all possible confounders (**Table 2**).

**Table 2:** Association between E2 levels and EAAC prevalence in postmenopausal woman.

ln (E2)		EAAC prevalence			
		Crude OR (95% CI)	*P*for trend	Adjusted OR (95% CI)	*P*for trend
Per unit decrease		1.54 (1.09, 2.18)	< 0.001	1.58 (1.02, 2.54)	< 0.001
Per tertile decrease	Tertile1	2.19 (1.18, 4.06)		2.55 (1.10, 5.92)	
	Tertile2	1.19 (1.07, 2.34)	< 0.001	1.31 (1.03, 2.57)	< 0.001
	Tertile3	Reference		Reference	

Crude model: adjusted for none. Adjusted model: adjusted for Age, Sex, Ethnicity, BMI, Drinking, Smoking, DM, Hypertension, Osteoporosis, OPD, RBC, WBC, Platelets, Albumin, Cr, TG, LDL-C, and HDL-C. OR, odds ratio; CI, confidence interval.

On a categorical scale, the crude model showed that Tertile1 and Tertile2 were 2.19[OR (95%CI), 2.19(1.18, 4.06)] and 1.19[OR (95%CI), 1.19(1.07, 2.34)] times higher risk of suffering from EAAC than Tertile3, respectively, and the adjusted model showed 2.55[OR (95%CI), 2.55(1.10, 5.92)] and 1.31[OR (95%CI), 1.31(1.03, 2.57)] times higher risk, respectively (**Table 2**).

### Subgroup and sensitivity analysis

**Figure 3:**
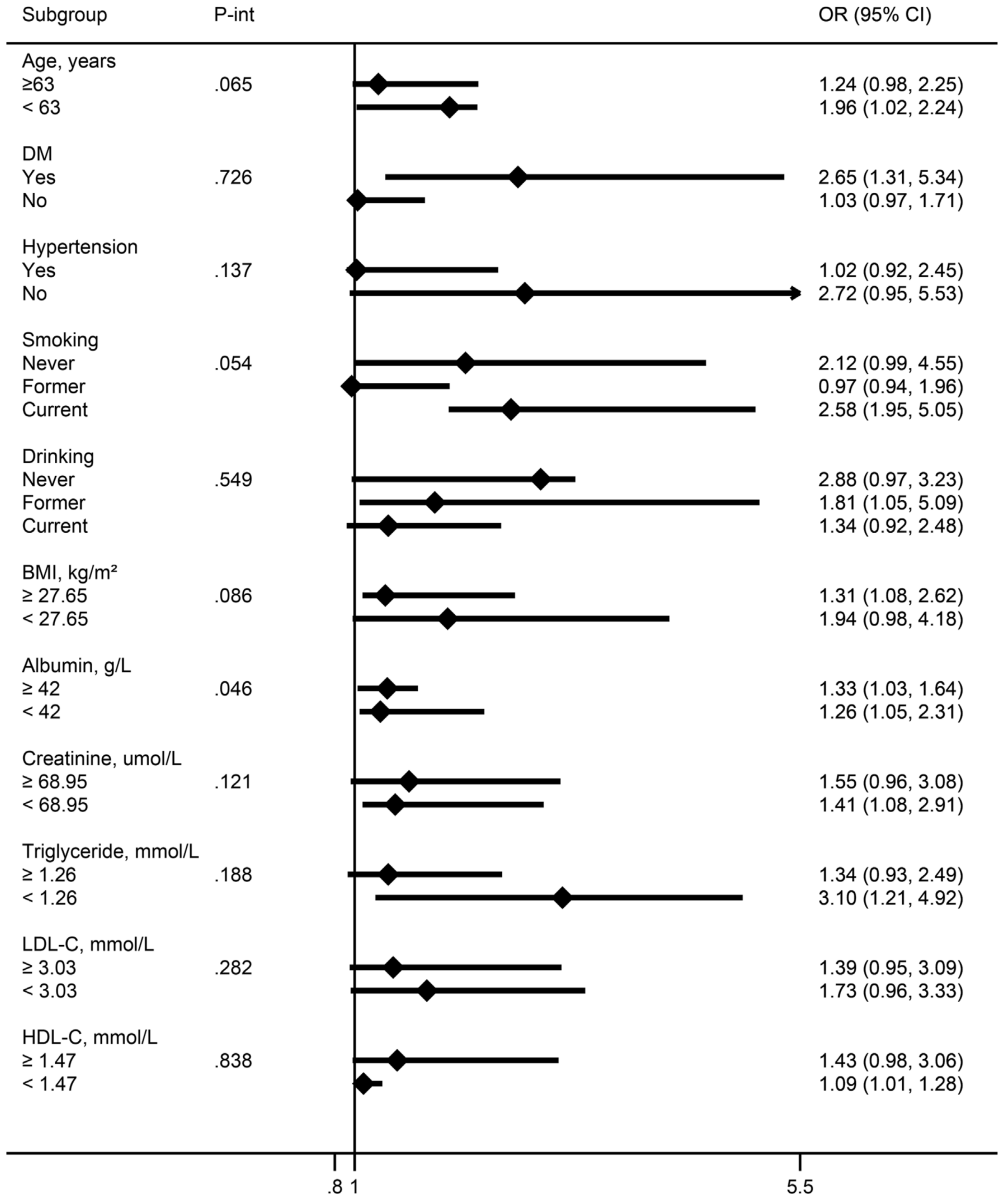
Association between E2 levels and EAAC prevalence in menopausal females in different subgroups [OR (95%CI), per unit ln(E2) decrease]. Continuous variables were grouped by median. All models were adjusted as in **Table 2**. OR, odds ratio; CI, confidence interval; P-int, P-interaction.

Subgroup analysis largely confirmed the associations between E2 levels and EAAC prevalence in postmenopausal women revealed in the current study across a broad spectrum of risk factors (**Figure 3**). Sensitivity analyses showed that the models in this study still yielded similar results when analyzed in women older than 45 years (**Table 3**).

**Table 3:** Association between E2 levels and EAAC prevalence in women older than 45 years.

ln (E2)		EAAC prevalence			
		Crude OR (95% CI)	*P* for trend	Adjusted OR (95% CI)	*P* for trend
Per unit decrease		1.82 (1.49, 2.22)	< 0.001	1.85 (1.43, 2.32)	< 0.001
Per tertile decrease	Tertile1	2.19 (1.18, 4.06)		2.55 (1.10, 5.92)	
	Tertile2	1.19 (1.06, 2.34)	< 0.001	1.36 (1.03, 2.57)	< 0.001
	Tertile3	Reference		Reference	

Crude model: adjusted for none. Adjusted model: adjusted for Age, Sex, Ethnicity, BMI, Drinking, Smoking, DM, Hypertension, Osteoporosis, OPD, RBC, WBC, Platelets, Albumin, Cr, TG, LDL-C, and HDL-C. OR, odds ratio; CI, confidence interval.

## Discussion

This study found that after adjusting for possible confounders, for per unit decrease in ln(E2), the risk of EAAC prevalence increased by 58%, and the risk of EAAC prevalence in tertile1 and tertile2 of the E2 level was 2.55 and 1.31 times higher than that in tertile3, respectively. To summarize, lower E2 levels were associated with a higher prevalence of EAAC.

Several previous studies have supported the findings of this study. J Nakao et al. found that serum E2 levels were lower in postmenopausal women with iliac artery calcification (8.4 ± 1.4 pg/mL) than in the control group (16.1 ± 1.6 pg/mL) (26), while the present study found that E2 levels were also significantly lower in the EAAC group (5.5 ± 4.6pg/mL) than in the non-EAAC group (8.6 ± 15pg/mL). Gyun-Ho Jeon et al. found higher coronary artery calcium accumulation (CACS) in postmenopausal women with lower serum E2 levels, independent of age and other coronary risk factors. Their results showed that after adjusting for possible confounders, the risk of CACS (>100) in subjects with E2 levels less than 20 pg/mL was 4-fold higher than in subjects with E2 levels greater than 20 pg/mL (27), while our multivariate analysis showed that subjects with an E2 range of 2.12-3.57 pg/mL had a 2.19-fold higher risk of EAAC than subjects with an E2 range of 7.06-38.4 pg/mL. The risk multiplier was higher than in the present study probably because the effects of diabetes mellitus and chronic kidney disease on arterial calcification were not taken into account. Samar R El Khoudary et al. found that E2 levels were negatively correlated with the extent of CAC in obese participants, but showed a significant positive correlation with the extent of CAC in non-obese participants (28). The conclusions of the above study are controversial because the included population was not postmenopausal and the potential E2 fluctuating effect from the menstrual cycle was not eliminated, but the interaction between E2 and obesity found in the study was consistent with that of the present study in the subgroup analyses. Although the study endpoints of the aforementioned studies were coronary artery calcification and iliac artery calcification rather than abdominal aortic calcification, arterial calcification has a common pathogenesis and is often systemic and segmental in nature. Thus, both our and previous studies have demonstrated a connection between E2 levels and arterial calcification, but the differences in the associations between E2 levels and different sites of calcification still need to be further demonstrated.

Two other studies showed findings that contradicted the present study and those mentioned above.

Erin D. Michos et al. found that there was no association between E2 levels and the presence or absence of AAC in postmenopausal women with subclinical atherosclerosis. The conflicting findings may be related to the selection of the study population and the definition of the study endpoints being different from the present study. Erin D. Michos et al. studied patients with subclinical atherosclerosis, who often have co-morbid chronic diseases such as diabetes mellitus, chronic kidney disease, hypertension, and vascular and valvular lesions, which are causally related to arterial calcification, potentially masking the true effect of E2 levels on AAC. In addition, the study’s definition of the presence (AAC>0) or absence (AAC=0) of AAC as a study endpoint may have attenuated the association between E2 levels and AAC. Because arterial calcification is a natural manifestation of aging, an AAC score <5 is considered to be the absence of significant calcification (19–21). The study may have included a large sample of subjects with AAC<5 who were considered to have undergone significant calcification. Pamela Ouyang et al. did a similar study in patients with subclinical atherosclerosis as did Erin D. Michos et al. The difference is that they did the association between E2 levels and carotid intima-media thickness (CIMT) and coronary artery calcification (CAC), so the same limitations as in the study by Erin D. Michos et al. exist.

Basic studies have shown that the cardiovascular protective effects of estrogen are attributable to estrogen-induced lipid changes on the one hand and to direct estrogen effects on the vasculature on the other hand (29–31). The mechanisms mediating the effects of estrogen on the vascular wall are not fully understood, but it has been reported that estrogen induces vasodilation by increasing the bioavailability of nitric oxide to endothelial cells (32). In addition, estrogen also prevents atherosclerosis by long-term inhibition of the vascular injury response through estrogen receptor-mediated changes in gene expression (32).

Another important aspect is that E2 plays a critical role in regulating bone metabolism and significantly impacts vascular calcification by influencing both osteogenesis and osteoclastogenesis processes. Traditionally, osteoporosis has been recognized as a condition primarily affecting postmenopausal women, largely due to the deficiency of E2 (33, 34). E2 regulates bone remodeling by modulating the production of cytokines and growth factors in the bone marrow and osteoblasts, a process mediated by the basic multicellular unit (BMU) (35). When E2 levels are deficient, BMU activity increases, leading to higher rates of osteoblast apoptosis and reduced osteoclast apoptosis. This imbalance shortens the bone formation period, preventing new bone from adequately filling the spaces left by resorbed old bone, resulting in significant bone loss and thus promoting the onset and progression of osteoporosis (36–38). A substantial body of evidence indicates that vascular calcification is a central event linking bone loss to cardiovascular risk (39). In particular, prospective epidemiological studies have shown a significant association between aortic calcification and lower bone density (40–42). Indeed, vascular calcification and osteoporosis share common risk factors and pathophysiological mechanisms, including the relationship between bone-derived proteins (e.g., osteoprotegerin and osteopontin) and vascular pathology, as well as the involvement of intercellular protein systems like the RANK/RANKL/OPG axis and Wnt signaling pathways (43). However, the interactions between bone loss and vascular calcification are complex, and their precise mechanisms and clinical significance remain unclear.

Estrone(E1), the predominant estrogen form in postmenopausal women, is known to be less potent than estradiol but still plays a critical role in maintaining bone density (44). The decline in E1 levels post-menopause is strongly associated with the onset and progression of osteoporosis (45). As estrone is integral to bone metabolism, its deficiency could contribute to both osteoporosis and the calcification of the abdominal aorta. This suggests that in postmenopausal women, low E1 levels might not only predispose them to osteoporosis but also exacerbate the risk of developing AAC (34, 46). On the other hand, studies suggest that E1 may be more likely than E2 to induce platelet aggregation, possibly due to its different regulatory effects on platelet surface receptors or cellular signaling pathways (47, 48). This characteristic makes E1 particularly significant in research related to cardiovascular diseases, especially when investigating the risk of thrombosis in postmenopausal women. Therefore, while our study primarily focused on the association between E2 levels and AAC, the role of E1 and its relationship with both osteoporosis and vascular calcification should not be overlooked. Future research should consider E1 levels as a variable of interest when exploring the links between bone health and cardiovascular outcomes in postmenopausal women. Such studies could provide deeper insights into the comprehensive role of estrogenic hormones in vascular health and further clarify the potential benefits of hormone replacement therapy that includes both E2 and E1 in preventing AAC and related cardiovascular diseases.

Several clinical trials have demonstrated a significant benefit of hormone replacement therapy for vascular calcification. Postmenopausal female participants treated with E2 got regressive CIMT (49). CIMT tended to progress in non-estrogen-treated users, whereas CIMT tended to regress in estrogen-treated users (50). Manson et al. recently showed in a randomized clinical trial that estrogen therapy reduced CACS as measured by cardiac CT (51). Lipid levels are considered important factors influencing vascular calcification (52). In subjects receiving estrogen/progestin therapy, HDL-C levels were elevated, while LDL-C levels were reduced (53, 54). However, another clinical trial showed that starting hormone replacement therapy many years after menopause (an average of 15 years) increases the risk of coronary heart disease (55).Thus, there is still controversy about whether estradiol has a beneficial effect on arterial calcification, and further confirmation from additional studies is needed.

The innovation of this study lies in its systematic exploration of the relationship between serum E2 levels and AAC, with a specific focus on postmenopausal women. While previous research has established a link between declining estrogen levels and increased cardiovascular risk, studies specifically addressing AAC as a subclinical marker have been relatively scarce. This study fills a critical gap in the literature and provides foundational data to further investigate the potential of estrogen replacement therapy in preventing AAC and associated cardiovascular diseases. However, this study has certain limitations. First, as a cross-sectional study, it can only demonstrate an association between E2 levels and AAC, without establishing a causal relationship. Second, the sample size is limited, which may affect the generalizability of the results. Third, not including osteoporosis as an inclusion criterion may limit our comprehensive understanding of the relationship between estrogen levels and AAC. Additionally, the study did not account for other factors that could influence AAC, such as lifestyle, dietary habits, other hormone levels, or long-term medication use, all of which could introduce potential confounding effects. Future research should consider longitudinal study designs with larger sample sizes to further validate these findings and explore the underlying mechanisms.

## Conclusion

This study found that a higher prevalence of AAC in postmenopausal women is closely associated with lower serum E2 levels. This finding supports the potential link between declining estrogen levels and increased cardiovascular risk, particularly in relation to the development of AAC. Our research further underscores the importance of E2 in maintaining cardiovascular health in postmenopausal women and suggests that monitoring E2 levels may aid in the early prevention and management of AAC and related cardiovascular diseases.

## Data Availability

The datasets presented in this study can be found in online repositories. The names of the repository/repositories and accession number(s) can be found below: the National Health and Nutrition Examination Survey (NHANES).
